# Asymptomatic kidney changes discovered during occupational health screening: age-dependent trends from a large cohort

**DOI:** 10.1093/ckj/sfag123

**Published:** 2026-08-07

**Authors:** Angela Stufano, Pietro Cirillo, Luigi De Maria, Giuseppe Delvecchio, Antonio Caputi, Ylenia Alberga, Francesca Di Serio, Maurizio Coggiola, Giuseppe Scarimbolo, Alessandro Mascolo, Simone Di Pace, Francesco Pesce, Marco Fiorentino, Giovanni Migliore, Luca De Nicola, Luigi Vimercati, Loreto Gesualdo

**Affiliations:** Department of Medical and Surgical Sciences, University of Foggia, Foggia, Italy; Nephrology, Dialysis and Transplantation Unit, Department of Precision and Regenerative Medicine and Ionian Area (DIMEPRE-J), University of Bari, Bari, Italy; Interdisciplinary Department of Medicine (DIM), University of Bari, Bari, Italy; Interdisciplinary Department of Medicine (DIM), University of Bari, Bari, Italy; Interdisciplinary Department of Medicine (DIM), University of Bari, Bari, Italy; Interdisciplinary Department of Medicine (DIM), University of Bari, Bari, Italy; Clinical Pathology Unit, University Hospital of Bari, Bari, Italy; University Hospital City of Health and Science of Turin, Turin, Italy; Nephrology, Dialysis and Transplantation Unit, Department of Precision and Regenerative Medicine and Ionian Area (DIMEPRE-J), University of Bari, Bari, Italy; Nephrology, Dialysis and Transplantation Unit, Department of Precision and Regenerative Medicine and Ionian Area (DIMEPRE-J), University of Bari, Bari, Italy; Nephrology, Dialysis and Transplantation Unit, Department of Precision and Regenerative Medicine and Ionian Area (DIMEPRE-J), University of Bari, Bari, Italy; Department of Translational Medicine and Surgery, Università Cattolica del Sacro Cuore, Rome, Italy; Nephrology, Dialysis and Transplantation Unit, Department of Precision and Regenerative Medicine and Ionian Area (DIMEPRE-J), University of Bari, Bari, Italy; Directorate-General for Communication, Ministry of Health, Rome, Italy; Nephrology Division, University “Luigi Vanvitelli” of Naples, Naples, Italy; Interdisciplinary Department of Medicine (DIM), University of Bari, Bari, Italy; Nephrology, Dialysis and Transplantation Unit, Department of Precision and Regenerative Medicine and Ionian Area (DIMEPRE-J), University of Bari, Bari, Italy

**Keywords:** age stratification, chronic kidney disease, early detection, eGFR, healthcare workers, occupational health, urinary albumin-to-creatinine ratio, young adults

## Abstract

**Background:**

The urinary albumin-to-creatinine ratio (uACR) and estimated glomerular filtration rate (eGFR) are key biomarkers for asymptomatic detection of chronic kidney disease (CKD), yet their combined use in preventive screening—particularly among young adults—remains uncommon. This study evaluates CKD markers in a large occupational cohort, emphasizing the early renal alterations observed in the 19–30-year age group and their distribution across older age strata.

**Methods:**

Between 2021 and 2024, 12 281 healthcare workers aged 19–71 years participated in an occupational health surveillance program at the University Hospital of Bari, Italy. CKD was evaluated using eGFR ( Chronic Kidney disease Epidemiology Collaboration ;CKD-EPI) and uACR from spot urine samples, with classification based on the KDIGO (Kidney Disease: Improving Global Outcomes) guidelines (2024). The results were stratified by age (19–30, 31–60, and >60 years) and comorbidities.

**Results:**

Overall, 6.7% of participants met the KDIGO criteria for CKD, with 6.5% exhibiting elevated uACR (>30 mg/g) and 0.2% demonstrating reduced eGFR (<60 mL/min/1.73 m^2^).

Young adults (19–30 years, *n* = 4139): 95.3% exhibited normal renal profiles (G1–G2/A1), while 4.7% showed increased albuminuria (A2–A3) despite preserved eGFR, indicating asymptomatic subclinical renal changes.

Middle-aged adults (31–60 years, *n* = 5245): 5.9% presented with mild renal impairment, characterized by elevated uACR or mild eGFR decline.

Older adults (>60 years, *n* = 2907): Only 84% remained in the lowest KDIGO prognostic category (G1–G2/A1), whereas 16% exhibited elevated uACR and/or reduced eGFR, consistent with age-related renal decline and a higher comorbidity burden.

Among the 74 individuals with severe albuminuria (A3), 10 underwent renal biopsy, which revealed IgA nephropathy, nephroangiosclerosis, lupus nephritis, and membranous glomerulonephritis (GN.

**Conclusion:**

Even in a predominantly healthy working population, one in 20 young adults demonstrated asymptomatic kidney alterations identifiable only through uACR screening. These findings underscore the importance of integrating both uACR and eGFR testing into occupational and preventive health programs to detect mild CKD in all age groups and promote kidney health preservation from early adulthood.

KEY LEARNING POINTS **What was known:**

- Chronic kidney disease (CKD) is highly prevalent and underdiagnosed worldwide, with limited awareness even among individuals with moderate disease.
- Early detection through biomarkers such as estimated glomerular filtration rate (eGFR) and urinary albumin-to-creatinine ratio (uACR) can prevent progression, yet their combined use in screening asymptomatic populations is uncommon.
- Preventive screening programs targeting healthy, working-age adults are rare, despite occupational and metabolic risk factors that may predispose them to CKD.

**This study adds:**

- Among 12 281 healthcare workers, 6.7% met CKD criteria, and asymptomatic kidney alterations were detected in 4.7% of young adults (19–30 years) with elevated uACR despite normal eGFR.
- Combined uACR and eGFR testing revealed subclinical renal changes that would have been missed by eGFR alone, confirming the diagnostic value of dual biomarker assessment.
- The findings highlight an age-related gradient in Kidney Disease: Improving Global Outcomes prognostic categories, underscoring the need for early, workplace-based CKD screening to promote timely prevention and intervention.

**Potential impact:**

- Integrating uACR and eGFR screening into occupational health programs can facilitate early detection of CKD, even among asymptomatic young workers.
- Early identification and intervention could reduce progression to kidney failure, decrease healthcare costs, and improve workforce health sustainability.
- The occupational screening model proposed could inform broader public health strategies for asymptomatic CKD prevention in the general population.

## INTRODUCTION

Chronic kidney disease (CKD) is a global emergency with around 800 million patients worldwide. It is a largely underdiagnosed disease that contributes significantly to morbidity and healthcare costs worldwide [1]. CKD is a silent and long-lasting asymptomatic disease [2]. The level of CKD awareness in the general population is still very low and patients are unaware of their condition, even when they are in a moderate/severe phase of kidney disease; similar low-grade awareness concerns well-known kidney failure risk factors such as diabetes and high blood pressure (BP) [3]. As a result, over 80% of CKD patients are unaware and not treated effectively by the new standard of care recommended by current guidelines (i.e. renin-angiotensin-aldosterone systen inhibition ;RAAS inhibition ) [1]. Early detection is critical, as identification and interventions at asymptomatic CKD stage can prevent progression to kidney failure and cardiovascular complications [4] and allow reducing the high costs of progression to the dialysis phase [5–7].

The urinary albumin-to-creatinine ratio (uACR) and estimated glomerular filtration rate (eGFR) are essential biomarkers for early CKD detection, yet remain underutilized in asymptomatic populations [8]. uACR, unlike dipstick tests, is unaffected by urine concentration and reliably indicates asymptomatic renal impairment. Despite KDIGO (Kidney Disease: Improving Global Outcomes) guidelines recommending their combined use, routine screening is hindered by limited awareness, cost barriers, poor implementation of clinical protocols, and a focus on symptomatic individuals [9, 10]. In Italy, early detection efforts have further declined due to the discontinuation of school-based and military medical screenings, exacerbating the gap between recommended practice and clinical reality.

Healthcare workers (HCWs), typically a healthy population of working-age adults, may face increased risk for CKD due to various occupational factors. Prolonged exposure to workplace stress, shift work, and nephrotoxic agents—such as certain medications and disinfectants—can impair kidney function [11]. The demanding nature of healthcare roles, especially in hospitals, may also lead to comorbidities like hypertension and metabolic disorders, further elevating CKD risk [12]. Therefore, targeted screening programs for HCWs are essential to enable early detection, mitigate modifiable risk factors, and promote occupational health.

Given the high economic and social burden of CKD—particularly in its advanced stages, which require costly interventions like dialysis or transplantation—preventive strategies through workplace screening could reduce long-term healthcare costs. Despite geographical variations in CKD prevalence, many at-risk individuals remain undiagnosed. Studies by Pesce *et al*. [13] and De Nicola *et al*. [14] demonstrate the effectiveness of targeted interventions in raising awareness and facilitating early diagnosis.

Furthermore, integrating CKD screening into workplace health programs aligns with broader efforts to promote healthy aging among the working population. As individuals spend a significant portion of their lives in professional settings, workplace-based health initiatives can serve as effective platforms for preventive care [15]. Encouraging early detection and proactive health management supports long-term well-being, reducing the risk of chronic conditions that impact both individual quality of life and workforce sustainability. This study aims to describe the prevalence and distribution of CKD markers—uACR and eGFR—across KDIGO categories in a large occupational cohort, with particular focus on young adults and age-related trends. The goal is to inform and enhance workplace-based screening strategies, promoting the routine use of these biomarkers to improve asymptomatic CKD detection and enable timely interventions, ultimately extending preventive efforts from occupational settings to the general population.

## MATERIALS AND METHODS

### Study design and population

This cross-sectional, observational study was conducted at the University Hospital of Bari from January 2021 to December 2024. The study population comprised all HCWs undergoing mandatory health surveillance as part of routine occupational medical assessments for fitness for work, following Italian labor law (Legislative Decree 81/08 and its amendments). All eligible HCWs were invited to participate in the study. No CKD stage was excluded a priori from the analytical framework. However, individuals with previously diagnosed advanced CKD (stage 4–5) are typically not eligible for active employment and are therefore underrepresented in occupational cohorts. In our population, a small number of participants were identified as having advanced CKD stages during routine screening. The low prevalence of advanced CKD stages (G4–G5) reflects the occupational nature of the cohort and the healthy worker effect, whereby individuals with severe chronic conditions are less likely to be actively employed. Inclusion criteria required that participants be actively employed at the hospital and undergo occupational health surveillance during the study period. HCWs were excluded if they had preexisting CKD stage 4–5, pregnancy, or recent hospitalization (within the last 3 months). Each participant provided written informed consent before data collection, and all clinical data were obtained during occupational medical visits.

During routine occupational health surveillance, participants underwent comprehensive clinical and laboratory assessments, including medical examination, anthropometry, BP, electrocardiogram, and metabolic profiling. Hypertension was defined as systolic BP ≥140 mm Hg and/or diastolic BP ≥90 mm Hg, or current antihypertensive therapy. Diabetes mellitus was defined using an integrated approach that included a documented prior clinical diagnosis reported in the medical history, current use of antidiabetic medication, and/or fasting plasma glucose levels >7.0 mmol/L. Weight and height were measured with minimal clothing using an OMRON BF511 scale and stadiometer, respectively; Body mass index (BMI) was calculated (kg/m^2^) and categorized as overweight (25–29.9), obese (≥30), or overweight/obese (≥25). Fasting blood (8.5 mL in serum-separating tubes) and morning spot urine (50 mL) were collected following standardized protocols. Blood samples were centrifuged at 3500 rpm for 10 min within 2 h. Serum and urine creatinine were measured enzymatically using the Abbott Alinity CC analyzer, traceable to IDMS. Calibration was performed with NIST reference materials: SRM 967 (serum) and SRM 914a (urine, purity 99.7 ± 0.3%).

Further analyses on the Alinity system included triglycerides (GPO-PAP method), total cholesterol (CHOD-PAP), HDL (homogeneous enzyme immunoinhibition), and LDL (homogeneous enzyme colorimetric). Full blood counts were performed using laser light scattering on the Sysmex XN-1000. Kidney function was assessed via plasma creatinine, eGFR (CKD-EPI formula), uACR, and protein-to-creatinine ratio. A detailed medical history was obtained, covering comorbidities such as diabetes, hypertension, dyslipidemia, cardiovascular, renal, urinary, and systemic disorders. All laboratory tests were conducted in a certified hospital laboratory under rigorous quality control protocols.

### Stratification and additional testing

Participants were stratified according to the KDIGO guidelines:

- eGFR-based CKD stages (G1–G5);
- Albuminuria stages (A1–A3, based on uACR levels).

eGFR was calculated using the CKD-EPI 2021 race-free equation. In particular, eGFR values (mL/min/1.73 m^2^) were categorized according to the KDIGO 2024 classification as follows:

G1: ≥90 mL/min/1.73 m^2^ (normal or high);

G2: 60–89 mL/min/1.73 m^2^ (mildly decreased);

G3a: 45–59 mL/min/1.73 m^2^ (mildly to moderately reduced);

G3b: 30–44 mL/min/1.73 m^2^ (moderately to severely decreased);

G4: 15–29 mL/min/1.73 m^2^ (severely decreased);

G5: <15 mL/min/1.73 m^2^ (kidney failure).

Albuminuria was classified based on the urine albumin-to-creatinine ratio (ACR) according to KDIGO guidelines into the following categories:

A1: <30 mg/g (normal to mildly increased);

A2: 30–300 mg/g (moderately increased);

A3: >300 mg/g (severely increased).

Abnormal albuminuria was defined as an ACR of ≥30 mg/g.

Subgroup analysis was conducted based on age groups and the presence of comorbidities, including diabetes, hypertension, obesity, and heart failure. Participants with uACR levels exceeding 30 mg/g were referred to the general practitioners as well as to the nephrologist consultation for additional diagnostic evaluations to confirm the CKD diagnosis, such as a 24-h urine collection to assess proteinuria and albuminuria, as well as measurement of creatinine clearance. In cases where uACR exceeded 300 mg/g, a second-level diagnostic investigations, such as renal ultrasound and kidney biopsy, were considered as part of the nephrological evaluation.

### Statistical analysis

Data were analysed using IBM SPSS Statistics, Version 24. Continuous variables were assessed for normality using the Shapiro-Wilk test. Data were presented as mean ± standard deviation (SD) or median with interquartile range, depending on the distribution. Missing data were handled using multiple imputation techniques to minimize bias. A *P*-value <.05 was considered statistically significant. Missing data accounted for ∼3.5% of the overall dataset. Multiple imputation was performed using 10 imputed datasets, including age, sex, eGFR, uACR, BMI, and major comorbidities (hypertension, diabetes, obesity, and heart failure) in the imputation model. Minor discrepancies between overall and age-stratified totals reflect missing age data in a small number of participants (*n* = 10; 0.08% of the cohort). Given the very limited proportion of missing values, no meaningful impact on the results or conclusions is expected.

### Ethical considerations

This study complied with the Declaration of Helsinki, ICH Good Clinical Practice, and national ethical regulations. The study was conducted as part of health promotion programs, and ethics committee approval was not required because all medical and instrumental examinations were performed in accordance with Italian laws concerning the protection of workers exposed to occupational risks (D. Lgs. 81/2008; D. Lgs. q 271/99). According to national regulations and institutional policies, secondary analyses of anonymized data collected during mandatory occupational health surveillance activities do not require formal ethics committee approval. All participants provided written informed consent, and data were anonymized and stored securely to protect confidentiality.

## RESULTS

### Study population and KDIGO risk stratification in the overall population

A total of 12 281 HCWs (ages 19–71 years) were enrolled between 2021 and 2024. General characteristics of the study population are shown in Table 1. According to the KDIGO heatmap classification (Table 2), the majority of participants fell into the low-risk category. Specifically, 93.3% (11 458 workers) were classified in the lowest KDIGO prognostic category (G1–G2/A1) (eGFR ≥60 ml/min/1.73 m^2^). Elevated uACR levels (>30 mg/g, corresponding to stages A2 and A3, G1–G3 KDIGO) were found in 6.5% of participants, while 0.2% had low eGFR (eGFR <60 ml/min with uACR <30 mg/g, classified as stage A1, G3 KDIGO). Only 0.6% of the population (74 workers) had a uACR >300 mg/g (A3 category), while the majority of workers showing a pathologic uACR >30–<300 mg/g (5.9%, 725 workers) fell into the A2 category. Few participants (25 workers) were categorized in the G3-A1 stage. Overall, 6.7% (823 workers) of the studied population met KDIGO criteria based on eGFR and/or uACR categories. Higher KDIGO prognostic categories were more frequently observed with increasing age and in participants with comorbidities. Finally, gender differences emerged in CKD prevalence: males showed a slightly higher proportion of individuals with eGFR stage G3 and normoalbuminuria (A1) compared to females (0.12% vs 0.08%), as well as a higher prevalence in the broader category of stages G1 to G3 with increased albuminuria (A2–A3) (3.4% vs 3.1%).

**Table 1:** General characteristics of the recruited population (*N* 12 281).

	Recruited subjects		
Variables	*N* (%)	Mean ± SD	Range
Age		39.6 ± 14.2	19.0–71.0
Gender			
-Male	5339 (43.47)		
- Female	6942 (56.53)		
eGFR (ml/min/1.73 m^2^)		103.20 ± 16.3	30–163.00
uACR mg/g		17.55 ± 100.2	0.00–2778.00
uPCR mg/g		95.65 ± 117.03	0.00–3507.00
eGFR-G3 and	25 (0.2)		
uACR/A1			
- Male	15 (0.12)		
- Female	10 (0.08)		
eGFR/G1–G3 and	798 (6.5)		
uACR/A2 e A3			
- Male	418 (3.4)		
- Female	380 (3,1)		
Subjects with heart failure	153 (1.24)		
Subjects with hypertension	308 (2.5)		
Subjects with diabetes	58 (0.47)		
Subjects with obesity (BMI > 30)	191 (1.55)		

**Table 2:** KDIGO classification according to uACR and eGFR concentrations in the overall population recruited from 2021 to 2024, considering the first urinary available value (*N*: 12 281).

eGFR\uACR	A1 *n* (%)	A2 *n* (%)	A3 *n* (%)	Overall n (%)
G1	10 046 (81.8)	602 (4.9)	49 (0.4)	10 697 (87.1)
G2	1411 (11.5)	111 (0.9)	13 (0.1)	1535 (12.5)
G3a	25 (0.2)	12 (0.1)	12 (0.1)	49 (0.4)
G3b	0 (0.0)	0 (0.0)	0 (0.0)	0 (0.0)
G4	0 (0.0)	0 (0.0)	0 (0.0)	0 (0.0)
G5	0 (0.0)	0 (0.0)	0 (0.0)	0 (0.0)
Overall	11 482 (93.5)	725 (5.9)	74 (0.6)	12 281 (100.0)

**Figure 1:**
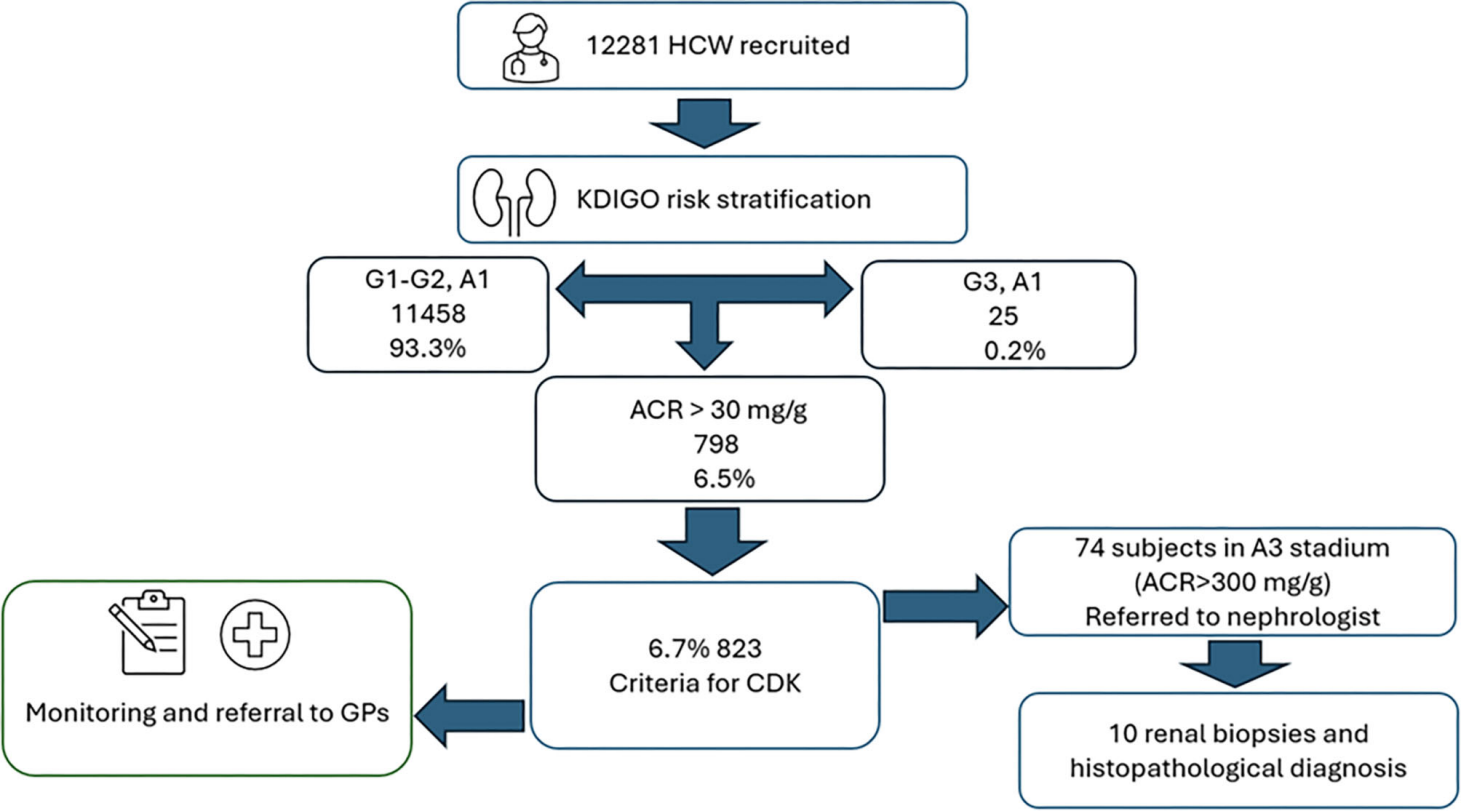
Flow chart of screening and clinical progression of the study population.

The 823 individuals meeting CKD criteria are now monitored every 6–12 months and have been referred to general practitioners and nephrologists. A flowchart showing the clinical evaluation of the study population is shown in Fig. 1.

### KDIGO risk stratification by age group

The age-stratified KDIGO analysis revealed marked differences in the distribution of CKD risk categories across age groups (Tables 3 –5). Among the youngest subgroup (aged 19–30 years, 4139 workers, mostly medical students attending the departments for internships or medical residents), 95.3% of individuals had normal kidney function and normoalbuminuria (G1–2, A1 categories), placing them in the lowest KDIGO prognostic category. In contrast, 4.7% of this population showed a moderate to high risk profile (A2–A3 categories). Specifically, 4.4% were classified as moderate (G1–2/A2 category), and only 0.3% showed severely increased albuminuria and were classified in the high-risk category (G1/A3). Virtually no individuals had a reduced eGFR, underscoring the expected renal health in this demographic. In contrast, the middle-aged group (31–60 years; 5245 workers) showed a modest but clinically meaningful shift in risk distribution. Although the majority (93.6%) remained in the G1–G2/A1 category, the prevalence of moderately increased albuminuria and mildly reduced eGFR (G1–G3a/A2) rose to 5.8%, and only 0.2% into G3a/A1, suggesting mild subclinical alterations in kidney function that may reflect age-related vascular or metabolic changes. Notably, the oldest subgroup (>60 years, 2907 workers) showed a substantial redistribution toward higher KDIGO risk categories. Only 84% of individuals remained in G1–2/A1, while 12.7% were classified as G1–3b/A2, 1.8% as G1–3b/A3, and 1.5% G3a–3b/A1. In terms of eGFR, 41.9% were classified as G2, and 2.4% as G3a or worse at the time of assessment.

**Table 3:** KDIGO classification for individuals aged 19–30 (first available values) (*N*: 4139).

eGFR\uACR	A1 *n* (%)	A2 *n* (%)	A3 *n* (%)	Overall *n* (%)
G1	3849 (93.0)	174 (4.2)	13 (0.3)	4036 (97.5)
G2	95 (2.3)	8 (0.2)	0 (0.0)	103 (2.5)
G3a	0 (0.0)	0 (0.0)	0 (0.0)	0 (0.0)
G3b	0 (0.0)	0 (0.0)	0 (0.0)	0 (0.0)
G4	0 (0.0)	0 (0.0)	0 (0.0)	0 (0.0)
G5	0 (0.0)	0 (0.0)	0 (0.0)	0 (0.0)
Overall	3944 (95.3)	182 (4.4)	13 (0.3)	4139 (100.0)

**Table 4:** KDIGO classification for individuals aged 31–60 (first available values) (*N*: 5245).

eGFR\uACR	A1 *n* (%)	A2 *n* (%)	A3 *n* (%)	Overall *n* (%)
G1	4185 (79.7)	257 (4.9)	20 (0.4)	4462 (85.0)
G2	718 (13.7)	41 (0.8)	10 (0.2)	769 (14.7)
G3a	5 (0.1)	5 (0.1)	5 (0.1)	15 (0.3)
G3b	1 (0.0)	0 (0.0)	0 (0.0)	1 (0.0)
G4	0 (0.0)	0 (0.0)	0 (0.0)	0 (0.0)
G5	0 (0.0)	0 (0.0)	0 (0.0)	0 (0.0)
Overall	4907 (93.5)	303 (5.8)	35 (0.7)	5245 (100.0)

**Table 5:** KDIGO classification for individuals aged >60 (first available values) (*N*: 2907).

eGFR\uACR	A1 *n* (%)	A2 *n* (%)	A3 *n* (%)	Overall *n* (%)
G1	1366 (47.0)	232 (8.0)	20 (0.6)	1618 (55.6)
G2	1078 (37.1)	131 (4.5)	11 (0.4)	1220 (42.0)
G3a	37 (1.2)	5 (0.2)	8 (0.3)	50 (1.7)
G3b	5 (0.2)	3 (0.1)	8 (0.3)	16 (0.6)
G4-5	0 (0.0)	0 (0.0)	3 (0.1)	3 (0.1)
Overall	2486 (85.5)	371 (12.8)	50 (1.7)	2907 (100.0)

### Clinical subgroups: heart failure, hypertension, diabetes, and obesity

Subgroup analyses (Supplementary Material, Tables S1–S4) revealed higher KDIGO prognostic category profiles among individuals with comorbidities. Workers with chronic heart failure (*N* = 153) showed increased prevalence of both albuminuria (A2, A3) and reduced eGFR (G3a–3b), in 15.6% and 4.5%, respectively. Similarly, those with hypertension (*N* = 308) showed increased prevalence of both albuminuria (A2, A3) and reduced eGFR (G3a–5), in 18.4% and 3.7%, and obesity (*N* = 191) displayed parallel distributions, with approximately 18.4% in A2–A3 stages and 3.7% in G3b–G5. The diabetic subgroup (*N* = 58) showed the highest burden of renal impairment: 27% had uACR >30 mg/g and 15.3% were in G3a–G5 stages. Among individuals presenting with elevated albuminuria (ACR >30 mg/g), a further comorbidity analysis revealed a high prevalence of overlapping metabolic and cardiovascular conditions. Specifically, 61 participants exhibited at least one additional comorbidity among diabetes, hypertension, or obesity (BMI > 30). Of these, 18 individuals had two or more of these conditions, and 3 participants presented with the full triad of diabetes, hypertension, and obesity alongside elevated ACR. These findings underscore the clustering of kidney dysfunction with other noncommunicable diseases, reinforcing the need for integrated screening strategies that simultaneously address renal, metabolic, and cardiovascular risk factors in occupational health programs.

### Subgroup with CKD undergoing kidney biopsy

Among individuals with severe albuminuria (A3), a limited subset underwent kidney biopsy, revealing heterogeneous histopathological findings; detailed results are reported in the Supplementary Material and are presented for descriptive purposes only. Among the 10 who had kidney biopsies, histopathological diagnoses included IgA nephropathy (IgAN), nephroangiosclerosis, lupus nephritis, and membranous glomerulonephritis. The average age of these subjects is 43.5 years, with an SD of 10.32 years. This indicates that the ages of the subjects are relatively spread out, ranging from 22 to 68 years. The gender distribution among these HCWs shows a slight male predominance, with six males (60%) and four females (40%). The mean uACR for these subjects is significantly elevated at 605.8 mg/g, with an SD of 338.86 mg/g. This wide range, from 147 to 1226 mg/g, suggests considerable variability in the severity of kidney dysfunction among the subjects.

### Histopathological features

The histopathological examination reveals a variety of kidney conditions among the subjects that underwent a kidney biopsy (*N* = 10). Three subjects were diagnosed with IgAN. The mean uACR for these subjects is 656 mg/g, with an SD of 433.8 mg/g, ranging from 421 to 1226 mg/g. Two subjects were found to have nephroangiosclerosis. The mean uACR for these subjects was 389.5 mg/g, with an SD of 342.9 mg/g, ranging from 147 to 632 mg/g. This finding suggests moderate to severe kidney damage. One subject was diagnosed with lupus nephritis, an inflammation of the kidneys caused by systemic lupus erythematosus. The uACR for this subject was 400 mg/g, indicating significant kidney involvement. Another subject had membranous glomerulonephritis. The uACR for this subject is notably high at 1089 mg/g, reflecting severe proteinuria. Minimal change disease was found in one subject. Lastly, one subject had a normal kidney biopsy at the light and immunofluorescence microscopy, yet the uACR for this subject was 593 mg/g. This suggests that despite the absence of histopathological abnormalities at the light and immunofluorescence microscopy, there is still significant proteinuria, which may warrant further investigation with electron microscopy.

## DISCUSSION

This work presents one of the largest cross-sectional analyses of kidney function conducted in a working-age population, comprising 12 281 HCWs aged 19–71 years old. Using the combined measurement of uACR and eGFR, we identified asymptomatic kidney alterations in 4.7% of young adults aged 19–30 years, a population segment rarely represented in previous CKD epidemiological studies, which generally enroll participants older than 35 years. The study provides two key and complementary insights: (i) In young adults, the presence of elevated uACR despite normal eGFR reveals subclinical or rare glomerular diseases, demonstrating the potential of uACR screening to uncover treatable early-stage pathology. (ii) In older adults, increased albuminuria and lower eGFR were more frequently observed in older participants and in those with cardiometabolic comorbidities , highlighting a clear age-dependent gradient of CKD risk within the same occupational cohort. Our findings emphasize the public-health relevance of implementing uACR and eGFR testing in occupational and preventive medicine frameworks. By detecting asymptomatic renal dysfunction in asymptomatic, working-age individuals—including those under 30 years old—such programs can bridge the existing diagnostic gap and align with KDIGO and World Health Organization recommendations for previously unrecognized, CKD detection and intervention.

These findings align with previous research emphasizing the importance of CKD screening in general populations [16, 17]. Pesce *et al*. [18] underscore the need for improved awareness and routine testing in primary care, reinforcing the utility of workplace-based screening initiatives. Overall, 6.7% (823 workers) of the studied population met the criteria for CKD. Higher KDIGO prognostic categories were increasingly prevalent with age and comorbidities. These results highlight the presence of mild-stage CKD markers even in a working population traditionally perceived as more health conscious [1]. Similarly, US occupational studies found higher CKD risk among healthcare support workers, likely due to long hours, stress, and environmental exposures [19]. Long working hours, in particular, have been identified as an independent risk factor for CKD, potentially driven by chronic stress, inadequate hydration, and limited time for physical activity [20]. While our study did not explore working conditions in depth, the demanding nature of healthcare roles—especially shift work and high-stress environments—may contribute to increased CKD risk.

The KDIGO age-stratified analysis showed a clear age-related progression in CKD risk. In participants over 60, a significant shift toward higher-risk KDIGO categories was observed, with more individuals exhibiting both increased albuminuria and reduced kidney function.

These findings are consistent with known physiological changes associated with kidney aging, such as nephron loss, diminished renal functional reserve, and increased vascular stiffness—all factors which contribute to microalbuminuria onset and declining eGFR. They emphasize age as a nonmodifiable but critical factor in CKD risk stratification. Importantly, these results also underscore the limitations of relying solely on eGFR, particularly in older adults, who may present with normal or mildly reduced eGFR but elevated albuminuria, a pattern strongly associated with cardiovascular risk [21].

Overall, the present findings should be interpreted as descriptive and hypothesis-generating, reflecting cross-sectional associations between kidney biomarkers, age, and comorbidities, rather than evidence of causality, disease mechanisms, or future risk.

From a public health standpoint, our study underscores the importance of assessing both eGFR and ACR when evaluating renal risk across age groups, as recommended by contemporary guidelines [8]. While younger adults may require less frequent monitoring, the absence of CKD awareness and routine screening among this group is concerning. Many younger individuals avoid or are excluded from routine screenings due to perceived low risk, yet early markers may still be present and go unnoticed. Our data suggest that failing to engage younger workers in preventive screening represents a missed opportunity for early detection and intervention. Expanding outreach and education around kidney health for this age group is essential [22].

This study reveals a prevalence of CKD among HCWs comparable to that observed in a 2008–2010 national Italian survey involving 7752 individuals from the general population [14]. Given that the mean age in the earlier study was 57 years—∼17 years older than the present HCW cohort—a lower CKD prevalence would have been anticipated. Although a definitive explanation remains elusive, the findings may suggest a greater burden of occupational or lifestyle-related risk factors in the HCW population. Mild-stage CKD is often clinically silent, yet its early identification significantly enhances the potential for cost-effective preventive strategies [9, 23]. Mandatory occupational health surveillance represents a promising platform for early detection, particularly among younger workers exposed to nephrotoxic risks [24]. Integrating routine assessments of eGFR and uACR into surveillance programs—especially for middle-aged and older HCWs—facilitates timely intervention [25, 26], supporting healthy aging and preserving a resilient healthcare workforce [27].

Among the ten individuals who underwent kidney biopsy, we observed notable variability in renal dysfunction and primary disease, highlighting the importance of individualized assessment. Prior studies, such as one on sugarcane workers in Cameroon, linked CKD to age and agrochemical exposure but lacked histological evaluation in high-albuminuria cases [28]. Our findings emphasize the value of early detection and pathology-based intervention. Biopsy gender distribution showed a slight male predominance (60%), aligning with evidence that men face higher CKD progression risk due to biological and behavioral factors [29–33].

Given its noninvasive and cost-effective nature, uACR testing should be embedded into occupational health frameworks to enhance early detection—especially for high-risk populations. [34, 35] The variability in uACR levels in our cohort further justifies the routine use of this test to identify individuals at various stages of dysfunction. Future studies should examine long-term outcomes and intervention strategies to support kidney health among workers, particularly those with known occupational exposures.

One striking finding is that none of the participants were aware of their CKD status. This highlights a major gap in awareness, consistent with the Croatian EH-UH 2 study, which reported that only 9.5% of CKD patients knew about their condition [36]. This widespread lack of awareness may be due to the silent nature of early CKD, limited screening in primary care, and low public education on kidney health. CKD is often overshadowed by its risk factors—hypertension, diabetes, and cardiovascular disease—leading to missed opportunities for early diagnosis and intervention. This study’s principal strength lies in its large, diverse cohort of HCWs, offering novel insights into early CKD markers within a younger, healthier population. The combined use of eGFR and uACR enhances diagnostic sensitivity, with albuminuria serving as a key early indicator. Biopsy-confirmed cases further validate findings, underscoring disease heterogeneity. Finally, although the present study does not directly assess the impact of screening on CKD prevention or clinical outcomes, the observed screening yield—particularly in young and asymptomatic individuals—supports the potential value of further longitudinal and interventional studies. Nonetheless, limited generalizability and missing work-related data represent key limitations.

A significant limitation of this study is its occupational nature. As expected, advanced CKD stages (G4–G5) were underrepresented, reflecting the healthy worker effect commonly observed in working populations. Individuals with severe CKD are less likely to remain in active employment and therefore less likely to be captured through occupational health surveillance. Consequently, our findings should not be interpreted as estimates of the population-level prevalence of advanced CKD, but rather as a description of CKD marker distribution within an actively working population. Additionally, since HbA1c was not systematically available in this occupational health examination survey, undiagnosed diabetes cannot be completely ruled out, which may have resulted in a slight underestimation of diabetes prevalence. Second, CKD classification in this health examination survey was based on a single measurement of eGFR and uACR, as commonly performed in large population-based studies; therefore, persistence of abnormalities over ≥3 months could not be assessed. In particular, isolated albuminuria—especially in young adults—may reflect transient or functional conditions rather than established CKD, and our findings should be interpreted as identifying CKD markers warranting further clinical confirmation rather than definitive CKD diagnoses.

In conclusion, this study demonstrates the feasibility and clinical value of uACR and eGFR screening for early CKD detection in the working population. Workplace-based programs can enable timely intervention, slowing disease progression and reducing complications. Policies that support regular uACR and eGFR testing among workers, including those typically excluded from general population screening, where older age is predominant, should be implemented to improve kidney health outcomes and preserve workforce capacity. Finally, the experience that would derive from the application of this model in the workplace should guide public health decisions regarding new prevention models for early CKD detection in the general population.

## Supplementary Material

sfag123_Supplemental_File

## Data Availability

The dataset used and analysed during the current study are available from the corresponding author on reasonable request.

## References

[1] GBD Chronic Kidney Disease Collaboration . Global, regional, and national burden of chronic kidney disease, 1990-2017: a systematic analysis for the global burden of disease study 2017. The Lancet. 2020;395:709–33. 10.1016/S0140-6736(20)30045-3 PMC7049905 32061315

[2] Mende CW. Chronic kidney disease and SGLT2 inhibitors: a review of the evolving treatment landscape. Adv Ther. 2022;39:148–64. 10.1007/s12325-021-01994-2 34846711 PMC8799531

[3] Kidney disease: a global health priority. Nat Rev Nephrol. 2024;20:421–3. 10.1038/s41581-024-00829-x 38570630

[4] Shlipak MG, Tummalapalli SL, Boulware LE et al. The case for early identification and intervention of chronic kidney disease: conclusions from a kidney disease: improving global outcomes (KDIGO) controversies conference. Kidney Int. 2021;99:34–47. 10.1016/j.kint.2020.10.012 33127436

[5] Chen JH, Chiu YW, Hwang SJ et al. Effect of nephrology referrals and multidisciplinary care programs on renal replacement and medical costs on patients with advanced chronic kidney disease: a retrospective cohort study. Medicine (Baltimore). 2019;98:e16808. 10.1097/MD.0000000000016808 31415394 PMC6831162

[6] McEwan P, Hafner M, Jha V et al. Translating the efficacy of dapagliflozin in chronic kidney disease to lower healthcare resource utilization and costs: a medical care cost offset analysis. J Med Econ. 2023;26:1407–16. 10.1080/13696998.2023.2264715 37807895

[7] Zhou J, Williams C, Staplin N et al. Effects of empagliflozin on quality of life and healthcare use and costs in chronic kidney disease: a health economic analysis of the EMPA-KIDNEY trial. EClinicalMedicine. 2025;85:103338. 10.1016/j.eclinm.2025.103338 40686671 PMC12274867

[8] Kidney Disease: Improving Global Outcomes (KDIGO) CKD Work Group . KDIGO 2024 clinical practice guideline for the evaluation and management of chronic kidney disease. Kidney Int. 2024;105:S117–314. 10.1016/j.kint.2023.10.018 38490803

[9] Cusick MM, Tisdale RL, Chertow GM et al. Population-wide screening for chronic kidney disease: a cost-effectiveness analysis. Ann Intern Med. 2023;176:788–97. 10.7326/M22-3228 37216661 PMC11091494

[10] Mahemuti N, Zou J, Liu C et al. Urinary Albumin-to-creatinine ratio in normal range, cardiovascular health, and all-cause mortality. JAMA Netw Open. 2023;6:e2348333. 10.1001/jamanetworkopen.2023.48333 38113044 PMC10731498

[11] Lan R, Qin Y, Chen X et al. Risky working conditions and chronic kidney disease. J Occup Med Toxicol. 2023;18:26. 10.1186/s12995-023-00393-3 37964292 PMC10644450

[12] Chen WC, Yang HY. Impaired kidney function among young healthcare workers with long working hours and night work. Scand J Work Environ Health. 2024;50:380–8. 10.5271/sjweh.4159 38567910 PMC11247656

[13] Pesce F, Bruno GM, Colombo GL et al. Clinical and economic impact of early diagnosis of chronic kidney disease in general practice: the endorse study. Clinicoecon Outcomes Res. 2024;16:547–55. 10.2147/CEOR.S470728 39130105 PMC11313497

[14] De Nicola L, Donfrancesco C, Minutolo R et al. Prevalence and cardiovascular risk profile of chronic kidney disease in Italy: results of the 2008–12 national health examination survey. Nephrol Dial Transplant. 2015;30:806–14. 10.1093/ndt/gfu383 25523453

[15] Stewart S, Kalra PA, Blakeman T et al. Chronic kidney disease: detect, diagnose, disclose—a UK primary care perspective of barriers and enablers to effective kidney care. BMC Med. 2024;22:331. 10.1186/s12916-024-03555-0 39148079 PMC11328380

[16] Muglia L, Di Dio M, Filicetti E et al. Biomarkers of chronic kidney disease in older individuals: navigating complexity in diagnosis. Front Med. 2024;11:1397160. 10.3389/fmed.2024.1397160 PMC11269154 39055699

[17] Brennan AT, Kileel EM, Khoza S et al. Prevalence and progression of chronic kidney disease among adults undergoing creatinine testing in South African public healthcare facilities: a study leveraging data from South Africa’s National Health Laboratory Service (NHLS). BMJ Public Health. 2024;2:e000799. 10.1136/bmjph-2023-000799 39698394 PMC11654635

[18] Pesce F, Pasculli D, Pasculli G et al. “The Disease Awareness Innovation Network” for chronic kidney disease identification in general practice. J Nephrol. 2022;35:2057–65. 10.1007/s40620-022-01353-6 35701727 PMC9584961

[19] Rubinstein S, Wang C, Qu W. Occupational risk and chronic kidney disease: a population-based study in the United States adult population. Int J Nephrol Renovasc Dis. 2013;6:53–9. 10.2147/IJNRD.S39522 23662070 PMC3647359

[20] Lee Y, Seo E, Mun E et al. A longitudinal study of working hours and chronic kidney disease in healthy workers: the Kangbuk Samsung Health Study. J Occup Health. 2021;63:e12266. 10.1002/1348-9585.12266 34382284 PMC8357818

[21] Musso CG, Oreopoulos DG. Aging and physiological changes of the kidneys including changes in glomerular filtration rate. Nephron Physiol. 2011;119:p1–5. 10.1159/000328010 21832859

[22] Berns JS. Routine screening for CKD should be done in asymptomatic adults selectively. Clin J Am Soc Nephrol. 2014;9:1988–92. 10.2215/CJN.02250314 25237073 PMC4220752

[23] Wish J, Al-Ghamdi SMG, Halimi JM et al. ‘Inside CKD’ multinational-microsimulation modelling insights into the increasing CKD burden. Kidney Int Rep. 2025;10:3356–68. 10.1016/j.ekir.2025.07.014 41141496 PMC12546715

[24] Chicas R, Mix J, Mac V et al. Chronic kidney disease among workers: a review of the literature. Workplace Health Saf. 2019;67:481–90. 10.1177/2165079919843308 31179873

[25] Farrell DR, Vassalotti JA. Screening, identifying, and treating chronic kidney disease: why, who, when, how, and what?. BMC Nephrol. 2024;25:34. 10.1186/s12882-024-03466-5 38273240 PMC10809507

[26] Levin A, Ahmed SB, Carrero JJ et al. Executive summary of the KDIGO 2024 clinical practice guideline for the evaluation and management of chronic kidney disease: known knowns and known unknowns. Kidney Int. 2024;105:684–701. 10.1016/j.kint.2023.10.016 38519239

[27] Carrillo-Alvarez E, Rodríguez-Monforte M, Fernández-Jané C et al. Professional competences to promote healthy ageing across the lifespan: a scoping review. Eur J Ageing. 2023;20:45. 10.1007/s10433-023-00794-7 37999781 PMC10673769

[28] Ekiti ME, Zambo JB, Assah FK et al. Chronic kidney disease in sugarcane workers in Cameroon: a cross-sectional study. BMC Nephrol. 2018;19:10. 10.1186/s12882-017-0798-9 29334929 PMC5769452

[29] Carrero JJ, Hecking M, Chesnaye NC et al. Sex and gender disparities in the epidemiology and outcomes of chronic kidney disease. Nat Rev Nephrol. 2018;14:151–64. 10.1038/nrneph.2017.181 29355169

[30] Neugarten J, Golestaneh L. Gender and the prevalence and progression of renal disease. Adv Chronic Kidney Dis. 2013;20:390–5. 10.1053/j.ackd.2013.05.004 23978543

[31] Whaley-Connell A, Nistala R, Chaudhary K. The importance of early identification of chronic kidney disease. Mo Med. 2011;108:25–8. 21462606 PMC6188457

[32] Bowe B, Xie Y, Li T et al. Particulate matter air pollution and the risk of incident CKD and progression to ESRD. J Am Soc Nephrol. 2018;29:218–30. 10.1681/ASN.2017030253 28935655 PMC5748906

[33] Levey AS, Becker C, Inker LA. Glomerular filtration rate and albuminuria for detection and staging of acute and chronic kidney disease in adults: a systematic review. JAMA. 2015;313:837–46. 10.1001/jama.2015.0602 25710660 PMC4410363

[34] Rossing P, Groehl F, Mernagh P et al. Estimated health economic impact of conducting urine albumin-to-creatinine ratio testing alongside estimated glomerular filtration rate testing in the early stages of chronic kidney disease in patients with type 2 diabetes. J Med Econ. 2023;26:935–43. 10.1080/13696998.2023.2235922 37439218

[35] Pesce F, Bruno GM, Colombo GL et al. Clinical and economic impact of early diagnosis of chronic kidney disease in general practice: the endorse study. Clinicoecon Outcomes Res. 2024;16:547–55. 10.2147/CEOR.S470728 39130105 PMC11313497

[36] Jelaković A, Radunović D, Josipović J et al. Prevalence, characteristics, and awareness of chronic kidney disease in Croatia: the EH-UH 2 Study. J Clin Med. 2024;13:6827. 10.3390/jcm13226827 39597972 PMC11594885

