# Supplementary material for: Asymptomatic kidney changes discovered during occupational health screening: age-dependent trends from a large cohort
Source: Clin Kidney J. 2026 Aug 7;19(8):sfag123. doi: 10.1093/ckj/sfag123 (PMC13460880; doi:10.1093/ckj/sfag123)
Supplement: Supplementary file 1 [file sfag123_supplemental_file.docx]

Table S1: KDIGO classification according to uACR and eGFR concentrations in workers with chronic **heart failure** (N:153)

|  | uACR | | |  |
| --- | --- | --- | --- | --- |
| eGFR | A1 n (%) | A2 n (%) | A3 n (%) | Overall n (%) |
| G1 | 83 (54.2) | 12 (7.8) | 2 (1.3) | 97 (63.4) |
| G2 | 43 (28.1) | 5 (3.3) | 1 (0.7) | 49 (32.0) |
| G3a | 2 (1.3) | 1 (0.7) | 1 (0.7) | 4 (2.6) |
| G3b | 1 (0.7) | 0 (0.0) | 1 (0.7) | 2 (1.3) |
| G4 | 0 (0.0) | 0 (0.0) | 0 (0.0) | 0 (0.0) |
| G5 | 0 (0.0) | 0 (0.0) | 1 (0.7) | 1 (0.7) |
| Overall | 129 (84.3) | 18 (11.8) | 6 (3.9) | 153 (100.0) |

Table S2: KDIGO classification according to uACR and eGFR concentrations in workers **with hypertension** (N:308)

|  | uACR | | |  |
| --- | --- | --- | --- | --- |
| eGFR | A1 n (%) | A2 n (%) | A3 n (%) | Overall n (%) |
| G1 | 165 (53.6) | 27 (8.8) | 2 (0.6) | 194 (63.0) |
| G2 | 84 (27.3) | 16 (5.2) | 2 (0.6) | 102 (33.1) |
| G3a | 0 (0.0) | 3 (1.0) | 2 (0.6) | 5 (1.6) |
| G3b | 2 (0.6) | 0 (0.0) | 3 (1.0) | 5 (1.6) |
| G4 | 0 (0.0) | 0 (0.0) | 0 (0.0) | 0 (0.0) |
| G5 | 0 (0.0) | 0 (0.0) | 2 (0.6) | 2 (0.6) |
| Overall | 251 (81.5) | 46 (14.9) | 11 (3.6) | 308 (100.0) |

Table S3: KDIGO classification according to uACR and eGFR concentrations in workers **with diabetes** (N:58)

|  | uACR | | |  |
| --- | --- | --- | --- | --- |
| eGFR | A1 n (%) | A2 n (%) | A3 n (%) | Overall n (%) |
| G1 | 29 (50.0) | 5 (8.6) | 3 (5.2) | 37 (63.8) |
| G2 | 10 (17.2) | 1 (1.7) | 1 (1.7) | 12 (20.7) |
| G3a | 1 (1.7) | 1 (1.7) | 2 (3.4) | 4 (6.9) |
| G3b | 2 (3.4) | 1 (1.7) | 1 (1.7) | 4 (6.9) |
| G4 | 0 (0.0) | 0 (0.0) | 0 (0.0) | 0 (0.0) |
| G5 | 0 (0.0) | 0 (0.0) | 1 (1.7) | 1 (1.7) |
| Overall | 42 (72.4) | 8 (13.8) | 8 (13.8) | 58 (100.0) |

Table S4: K-DIGO classification according to uACR and eGFR concentrations in workers **with obesity** (BMI> 30) (N:191)

| eGFR / ACR | A1 n (%) | A2 n (%) | A3 n (%) | Overall n (%) |
| --- | --- | --- | --- | --- |
| G1 | 103 (53.9) | 17 (8.9) | 1 (0.5) | 121 (63.4) |
| G2 | 52 (27.2) | 10 (5.2) | 1 (0.5) | 63 (33.0) |
| G3a | 0 (0.0) | 2 (1.0) | 1 (0.5) | 3 (1.6) |
| G3b | 1 (0.5) | 0 (0.0) | 2 (1.0) | 3 (1.6) |
| G4 | 0 (0.0) | 0 (0.0) | 0 (0.0) | 0 (0.0) |
| G5 | 0 (0.0) | 0 (0.0) | 1 (0.5) | 1 (0.5) |
| Overall | 156 (81.7) | 29 (15.2) | 6 (3.1) | 191 (100.0) |

Table S5: General characteristics of the subjects underwent to kidney biopsy (N 10)

|  | Recruited subjects | | |
| --- | --- | --- | --- |
| Variables | N(%) | Mean ± SD | Range |
| Age | 10 (0.11) | 43.5± 10.32 | 22-68 |
| Gender  -Male  -Female | 6 (60)  4 (40) |  |  |
| u-ACR mg/g |  | 605,8 ± 338,86 | 147-1226 |
| AER (albuminuria excretion rate) (mg/24h) |  | 643 ± 363,8 | 80-1200 |
| u- ACR mg/g > 30 | 10 (100) |  |  |

Table S6: Histopathological features and uACR at kidney biopsy time (N 10)

| Kidney Biopsy |  | | |
| --- | --- | --- | --- |
| Histopathological features | Number | uACR mg/gr (Mean ± SD) | Range mg/gr |
| IgAN | 3 | 656 ± 433,8 | 421 – 1226 |
| Nephroangiosclerosis | 2 | 389,5 ± 342,9 | 147 – 632 |
| Lupus Nephritis | 1 | 400 |  |
| Membranous Glomerulonephritis | 1 | 1089 |  |
| ESKD | 1 | 730 |  |
| Minimal Change Disease | 1 | 277 |  |
| Normal Kidney | 1 | 593 |  |
